# Rapidly Rising Preference among New-entry Medical Students for Using Generative Artificial Intelligence in Reflective Reports

**DOI:** 10.31662/jmaj.2024-0390

**Published:** 2025-03-28

**Authors:** Chihiro Kawakami, Osamu Nomura, Miyuki Takahashi, Ritsuki Takaha, Takayasu Ito, Hiroaki Ushikoshi, Takuya Saiki

**Affiliations:** 1 Medical Education Development Center, Gifu University, Gifu, Japan; 2 Department of Community-Based Co-Creative Hida-Takayama Health Professions Education, Gifu University, Gifu, Japan; 3 Center for Clinical Training and Career Development, Gifu University Hospital, Gifu, Japan; 4 Center for Regional Medicine, School of Medicine, Gifu University, Gifu, Japan

**Keywords:** artificial intelligence (AI), medical students, arts-based education, community-based inquiry learning, writing assignments

## Abstract

**Introduction:**

As artificial intelligence (AI) continues to proliferate, it becomes imperative that medical students are not only instructed in the use of AI but also afforded regular opportunities to interact with it throughout their medical education. In 2023 and 2024, an art-based reflective reports assignment was implemented among new-entry students of the medical program at a university in Japan. The assignment required students to use generative AI within their submissions. The objective of this study was to assess the perceptions of these students regarding the use of generative AI in their reflective reports over the course of two academic years.

**Methods:**

First-year medical students were tasked with submitting an art-based reflective report about their experiences during the community-based inquiry learning session. The assignment was a component of their reflective competencies assessment. We conducted a survey of the 2023 and 2024 student cohorts and examined their perceptions of using generative AI using a five-point Likert scale. We compared the survey responses of the two cohorts using the t-test.

**Results:**

The participants exhibited a notably higher mean score for positive perceptions toward generative AI in 2024 compared with 2023 (3.8 versus 2.9 points; p < 0.01). In addition, the proportion of participants who expressed a preference for using generative AI was significantly higher in 2024 than in 2023 (32.0% versus 17.4%; p = 0.02).

**Conclusions:**

A substantial and rapid increase has been observed in the proportion of medical students indicating a preference for using generative AI. The joint adoption of generative AI by medical students and faculties signifies a significant and urgently required development in AI use within medical education.

## Introduction

The rapid development of artificial intelligence (AI) has led to the expectation that AI will be used in medical care to enable patients to receive standardized care regardless of location, optimize healthcare professionals’ workflows, reduce workload, and develop new diagnostic and treatment methods ^(1)^. As the use of AI continues to expand ^(2)^, it is imperative that medical students have the opportunity to not only learn how to use AI but also regularly interact with it throughout their medical education. Generative AI is a technology that outputs new data and information from the vast quantities of data that machines have learned, thereby creating new ideas and content such as text, images, and movies ^(3)^. The potential of generative AI is being investigated in educational settings, including those at the post-secondary level.

In assessing students in higher education, the most prevalent alternative to the conventional paper-and-pencil examination has been written assignments such as reflective essays and reflective reports. A limited number of instructors review these submissions, and it is a laborious task to fairly evaluate the work of many students, sometimes numbering in the hundreds. Moreover, there is concern that the evaluation process may be susceptible to bias in favor of students proficient in written communication, regardless of the assignment content’s quality. Additionally, providing formative feedback on assignments to all students presents a practical challenge ^(4)^. The advent of generative AI has given rise to concerns surrounding its ability to be used to generate written assignments ^(5)^. In response, Japan’s Ministry of Education, Culture, Sports, Science and Technology (MEXT) has issued guidelines delineating the appropriate use of generative AI in student learning ^(6)^. This guideline delineates the typical scenarios in which generative AI can be effectively implemented to facilitate self-directed learning among students. These scenarios include brainstorming to generate ideas, organizing discussion points, efficient information retrieval, language editing and translation, and supplementary programming.

The incorporation of arts-based education into the undergraduate medical education curriculum has been a global phenomenon, with medical schools across the globe adopting this approach ^(7)^. The traditional medical education curriculum has historically incorporated an “art” component for assessing students’ structural understanding, particularly in the domains of anatomy, histology, and pathology ^(8)^. Some novel arts-based courses have been reported in which students analyzed a variety of artistic works such as paintings, photographs, and poetry. The objective of these initiatives is to cultivate in medical students a set of non-technical competencies, including empathy, communication, and ethical reasoning ^(9)^. The arts can be incorporated into students’ reflective reports, as the evaluation and creation of art facilitate medical students’ self-awareness and reflection, which cannot be assessed by simply writing essays. Art-based reflective reports are defined as reports that incorporate artistic works, such as illustrations and images, which convey the experience gained through learning opportunities, along with reflective descriptions of the works. While the application of art-based reflective reports is a novel approach to assessing non-classical competencies, it is not without limitations. The quality of the art produced by students may not directly reflect the quality of their reflections; thus, it is imperative to ensure that evaluation is objective and devoid of subjective judgments by teachers regarding the quality of students’ work. One potential solution to the issues in assessing different aspects of medical students’ competencies via reflective reports is to assign an art task that requires students to use generative AI ^(10)^. This approach can not only increase opportunities for students to use generative AI for academic purposes but also capitalize on generative AI’s capacity to generate images. Nevertheless, there is a paucity of research examining medical students’ evolving perceptions of generative AI and its use in reflective reports.

In 2023 and 2024, we implemented an art-based reflective reports assignment among new-entry students of the medical program at a university in Japan. The assignment required students to use generative AI within their submissions. Our objective in this study was to assess the perceptions of these students regarding the use of generative AI in their reflective reports over the course of two academic years and to identify any notable differences in their responses. Our hypothesis posits that medical students’ perceptions regarding the use of generative AI may have become more positive in 2024 compared with 2023. This shift could be attributed to the enhanced usability of generative AI and its growing social acceptability for academic purposes.

## Materials and Methods

### Setting

The study setting was a community-based inquiry learning session for first-year medical students. During the two-month program, students learned about community medicine by visiting and observing nine healthcare facilities, such as hospitals, clinics, and nursing homes, in groups of ten students each.

### Context

**Figure 1.**
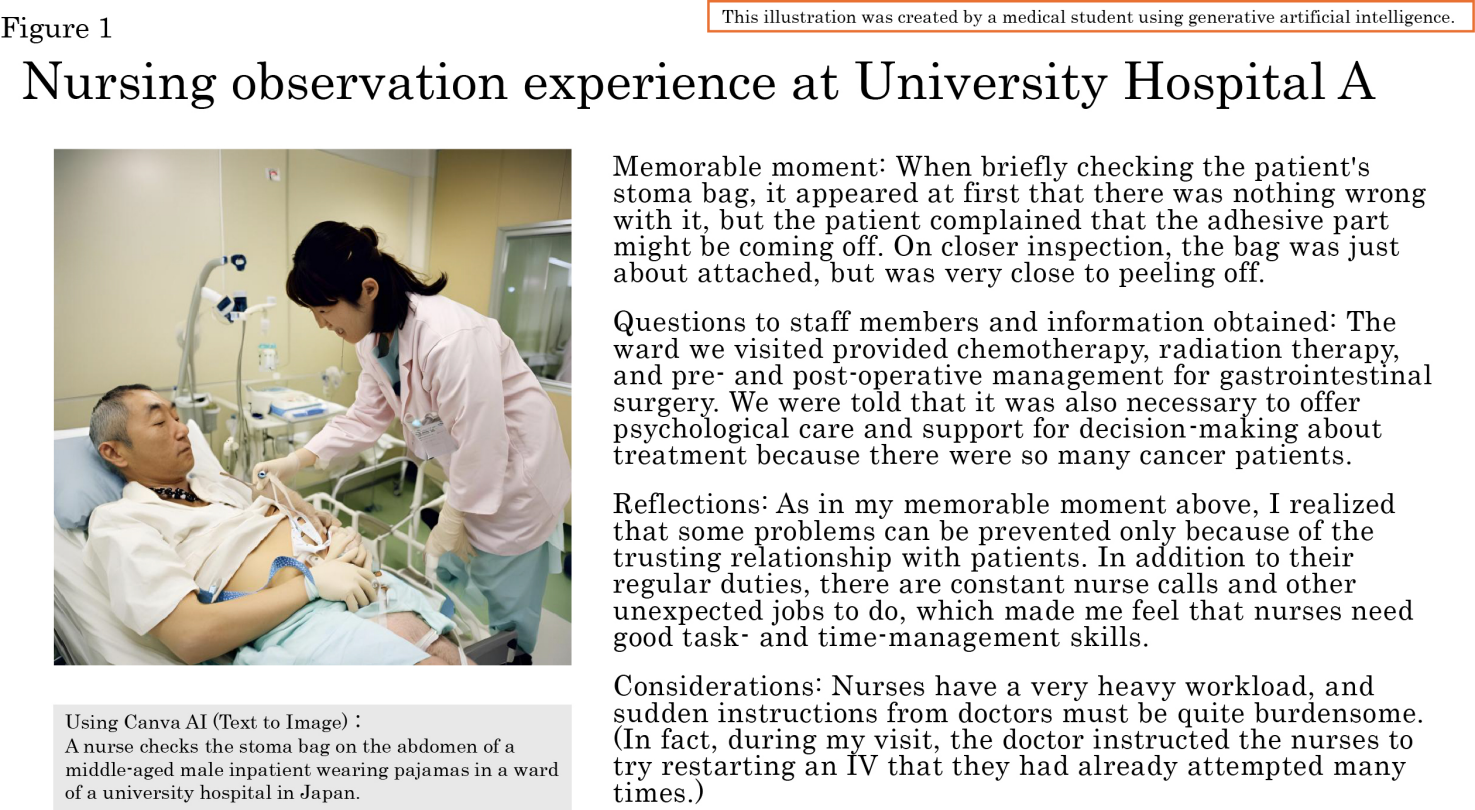
A sample page of an art-based reflective report submitted by a student. The student consented to the report being used in this paper.

We used an art-based reflective report submission to assess the reflective competencies of the students. Each student was required to submit a one-page artwork for their observation experience at each healthcare facility (i.e., nine pages in total). The students were tasked with visually expressing memorable moments and experiences from the observation period. The submissions could take the form of illustrations generated by generative AI, hand-drawn illustrations, or photographs taken by the students, but students were required to employ generative AI to create the artwork for at least one page of their reflective reports. Additionally, they were required to provide a written reflection, which was to be included on the same page as the artwork. A sample page of a submitted reflective report is presented in Figure 1.

The evaluation of the reports was conducted on the basis of the following criteria: The report should contain new discoveries about oneself that were made during the observation period, include a description of one’s own actions during the observation, and appropriately express and reflect upon memorable moments, while the content of the reflective essay should match the artwork used to express the memorable moment.

### Participants

Among the medical students who attended the courses, 109 (92.4%) and 100 (89.3%) students participated in this study in 2023 and 2024, respectively. The participants responded to an online survey after completing the course assignment.

### Measurement

The survey comprised the following three items: (1) whether the student had previous experience using generative AI for image creation (Have you created illustrations using generative AI before this assignment? Yes/No); (2) whether the student had formed a positive view of using AI in this way (Did you enjoy using generative AI for this assignment? Five-point Likert scale: 1 = not at all, 5 = a lot); and (3) whether the student preferred using AI over other methods to create his/her art-based submission (Which method do you prefer for creating your learning portfolio submission? Generative AI/Other methods including photos and drawings).

### Analysis

We used the t-test to compare the survey responses of the 2023 and 2024 cohorts. SPSS version 28.0 was used for the analysis.

## Results

The proportion of students who had previous experience of using generative AI to create images did not differ significantly between the 2023 and 2024 cohorts (8.3% and 15.0%, respectively; p = 0.10). In contrast, in response to the question of whether they had a positive view of using generative AI for image creation, students gave a significantly higher mean score in 2024 than in 2023 (3.8 versus 2.9 points; p < 0.01). Similarly, the proportion of students who stated a preference for using generative AI was significantly higher in 2024 than in 2023 (32.0% versus 17.4%; p = 0.02).

**Table 1.** Students’ Survey Responses.

	2023 (n=109)	2024 (n=100)	p-value
Previous experience of using generative AI, n, (%)	9 (8.3)	15 (15.0)	0.095
Positive view of using generative AI, Mean (SD)	2.9 (1.2)	3.8 (1.0)	<0.001
Preference for using AI over other methods, n (%)	19 (17.4)	32 (32.0)	0.016

## Discussion

The present study examined the experiences and perceptions of medical students regarding the integration of generative AI into their reflective reports. Over the course of the study, we observed a notable increase in the proportion of students who expressed a preference for using generative AI. While several factors may have caused this result, it is hypothesized that the timing of Chat Generative Pre-trained Transformer (ChatGPT)’s release (November 2022) is the primary factor contributing to the observed results. First-year students in 2023 may have had limited time and opportunity to use generative AI, such as ChatGPT, prior to the start of course activities in Spring 2023. Conversely, students in 2024 are expected to have had an additional year of exposure to generative AI.

The current findings align with the international literature from general higher education and undergraduate medical education. A single university survey conducted in the United Kingdom reported that the majority of undergraduate and graduate students in 2024 were familiar with the use of generative AI ^(11)^. In addition, a multicenter study published in 2024 to assess Chinese medical students’ acceptance of generative AI tools showed that ChatGPT was the most commonly used tool for academic purposes and that knowledge of generative AI significantly predicted students’ measured intention to use generative AI for their studies ^(12)^. These recent studies have shown that young generations around the world have positive perceptions about using generative AI to support their academic work. This may be attributable to the fact that contemporary university students have attained fundamental Information Technology (IT) competencies during their primary and secondary education, preceding their entry into higher education. In 2022, MEXT published the revised Model Core Curriculum for Medical Education in Japan ^(13)^, which newly incorporated IT as one of its core competencies. The IT competency states that medical students should recognize the impact of ongoing technological advancements and use information science and emerging technologies, such as AI, in their learning and future clinical practice. Because the subjects of the current study were first-year medical students, they would have only had several months of core-curriculum-based IT education prior to participating. It is therefore posited that the pre-university IT education students received exerted a more significant influence on the current findings than the IT education they were receiving at medical school.

The Model Core Curriculum can play an effective role in further developing the fundamental IT competencies that medical students acquire in primary and secondary education. Nonetheless, the promotion of innovative IT, including generative AI for academic tasks, may lead to students developing misconceptions regarding its application. These misconceptions might encompass the belief that generative AI is appropriate for use toward all class objectives, including the completion of assignments. Consequently, it is the responsibility of instructors to closely supervise students who may engage with generative AI in excessive or overdependent ways ^(14)^. Instructors often use AI detection tools to investigate whether students have employed generative AI in their assignments. These detectors have the potential to erroneously identify reports authored by students as AI-generated, particularly in cases where the students possess inadequate medical knowledge ^(15)^. This is a significant concern when assessing the written assignments of medical students. However, reflective reports that incorporate illustrations created by generative AI are not affected by this issue, as the quality of the illustrations generated by generative AI is not influenced by the extent of medical knowledge present, which may be a benefit of using art-based reports.

The present study has revealed that the proportion of medical students who have previous experience using generative AI for image creation remains low and did not increase significantly between 2023 and 2024. This finding aligns with the results of a national survey examining the usage habits of generative AI among Japanese university students, which reported that approximately 35% of students engage with generative AI on a regular basis, but only 10% of those who use generative AI create images using the technology ^(16)^. However, our findings indicate a notable increase in the proportion of students expressing a preference for using generative AI over just two consecutive years. A further national study has demonstrated that approximately 50% of university students have used generative AI for the purpose of composing reports and/or translating language for their academic courses ^(17)^. This may be indicative of the fact that university students are already using generative AI in their studies. It is therefore no longer effective to ban the use of generative AI for today’s digitally native students, and furthermore, it is essential to provide students with the opportunity to understand how these technologies can be employed toward productive learning ^(18)^.

The present study is not without its limitations. First, it was conducted within a single course at a single medical school in Japan. Therefore, it is uncertain whether the current findings can be applied to other educational settings. However, there is a possibility that elements of our results may be applicable to other educational contexts that share similar teaching and learning environments. Second, this study was conducted in an educational setting that employed generative AI to develop students’ IT skills. However, the effectiveness of incorporating generative AI into the course was not evaluated, such as by comparing it with a course that did not utilize AI. A holistic program evaluation study is needed to overcome these limitations.

In conclusion, the proportion of medical students stating a preference for using generative AI increased significantly and rapidly. The co-adoption of generative AI by medical students and faculties represents a significant and urgently needed development.

## Article Information

### Conflicts of Interest

None

### Sources of Funding

This work was funded by the “Near-peer teaching in community oriented medical education based on onsite and virtual learning integrated with anthropology” project, supported by the “Establishing Bases for Fostering Medical Personnel in the Post-COVID Era” project of the Ministry of Education, Culture, Sports, Science and Technology in Japan.

### Acknowledgement

We thank Dr Hiroaki Kawashiri, Dr Tadao Goto, Dr Rintaro Imafuku, Dr Kaho Hayakawa, Dr Kazuhiko Fujisaki, Ms Kyoko Kubota, Ms Reiko Fujii, and Ms ALATANNABUQI for their support in data collection. We also thank Mr Oliver Stanyon for editing the manuscript.

### Author Contributions

CK: Conceptualization, Investigation, Methodology, Writing - Original Draft Preparation.

ON: Formal analysis, Writing - Original Draft Preparation.

MT & RT: Data Curation, Writing - Review & Editing.

TS: Project Administration, Supervision, Writing - Review & Editing.

### Approval by Institutional Review Board (IRB)

This study was conducted with the approval of the Gifu University Institutional Review Board (No. 2022-215: Approved on June 20, 2022).

### Patient Consent for Publication

Not applicable.

### Informed Consent

Informed consent has been obtained from all participants.
